# The role of parental mentalizing on the link between parental depression and child emotion regulation

**DOI:** 10.1111/papt.12563

**Published:** 2024-12-10

**Authors:** Mor Keleynikov, Dana Lassri, Noga Cohen, Joy Benatov

**Affiliations:** ^1^ Department of Special Education, Faculty of Education University of Haifa Haifa Israel; ^2^ The Paul Baerwald School of Social Work and Social Welfare The Hebrew University of Jerusalem Jerusalem Israel; ^3^ The Edmond J. Safra Brain Research Center for the Study of Learning Disabilities University of Haifa Haifa Israel

**Keywords:** emotion regulation, emotion socialization, parental depression, parental mentalizing, parental reflective functioning, preschoolers

## Abstract

**Background:**

Parental depression has consistently been shown to impact child's emotion regulation (ER), with limited research on risk and protective factors, especially in preschoolers. Grounded in Morris and colleagues' model of emotion socialization, this study addresses this gap by examining the mediating and moderating roles of parental mentalizing (reflective functioning).

**Aims:**

We aimed to explore whether pre‐mentalizing modes mediate the link between parental depressive symptoms and the child's ER tendencies and whether parental interest and curiosity and parental certainty about mental states can buffer this link.

**Materials & Methods:**

The study sample included 732 parents (91% females) of children aged 3–7 years. To assess parental mentalizing, the Parental Reflective Functioning Questionnaire was used. Child ER skills were assessed with the Emotion Regulation Checklist, and depression was assessed with the Patient Health Questionnaire.

**Results:**

Findings demonstrated a negative link between parental depressive symptoms and children's ER skills, mediated by heightened parental pre‐mentalizing modes. Contrary to expectations, parental interest and curiosity did not moderate this link, but parental certainty about mental states emerged as a protective factor, weakening the link between depressive symptoms and child ER skills.

**Conclusion:**

The results contribute to the understanding of ER development, particularly in the context of parental depressive symptoms, emphasizing parental mentalizing as a pivotal factor within this intricate dynamic.

## INTRODUCTION

During preschool, children gradually learn to regulate their emotions, a process vital for many dimensions of children's development (Feng et al., 2008; Kopp, 1989). Emotion regulation (ER) is often defined as a set of processes that enable individuals to monitor, evaluate and modify their emotional reactions (Gross, 1998). Children's ER skills significantly impact various aspects of functioning and are considered a transdiagnostic risk factor for the development of various psychiatric disorders (Lynch et al., 2021). For example, research indicates that deficits in ER are associated with increased behavioural problems, challenges in peer relationships and mental health issues including depression and anxiety (see for reviews: Sala et al., 2014; Zeman et al., 2006). Conversely, children with good ER skills tend to exhibit better academic performance and enhanced social abilities (Graziano et al., 2007; Harrington et al., 2020). Recognizing the importance of ER for children's adjustment and functioning highlights the need to identify the factors that contribute to the development of ER skills.

During preschool age, children learn to handle their emotions in different ways. At first, they rely on their parents to regulate emotions, but as they grow, they become more differentiated and learn how to regulate themselves (Feng et al., 2008; Montroy et al., 2016). This development involves mastering a diverse set of behavioural strategies, enabling them to adeptly manage their emotions (Cole et al., 2009). During this time, the child learns how to regulate emotions, without the need for adult assistance. According to Morris model of emotion socialization (2007), the process of ER in this age heavily depends on both personal factors such as temperament and genetic dispositions, as well as external factors that mostly include parental‐related factors. These factors include parental ER tendencies, parenting practices and psychopathology among parents.

### Parental depression and children emotion regulation

When discussing parental psychopathology and its effect on children's ER, a significant area of research focusses on the impact of parental depression (Wu et al., 2020).

In their review, Morris et al. (2007) suggest that parents shape children's ER across three crucial areas. First, children acquire ER skills through observation of their parents. Past research indicates that individuals experiencing depression often struggle with effective ER and parental mentalizing (the ability to infer behaviour as an expression of mental states), and therefore might provide maladaptive models of emotional expression and regulation as well as impaired capacity to self‐reflect to their children (Granat et al., 2017; Schultheis et al., 2019; Visted et al., 2018). As children learn to regulate their emotions by watching and imitating their parents, children with depressed parents might learn unhealthy ways to manage their emotions (Keleynikov et al., 2023; Silk et al., 2006; Wu et al., 2020). Second, the family's emotional atmosphere, indicated by attachment quality, parenting styles and marital relationship quality, impacts children's ER. Parental depressive symptoms could potentially impact each of these domains, shaping children's ER abilities as a result. For example, parents experiencing depression frequently exhibit diminished positive affect and responsiveness during interactions with their children (Campbell et al., 2004). These parents may also demonstrate increased negativity and hostility as well as reduced sensitivity and responsiveness in face‐to‐face interactions, thereby elevating the risk of self‐regulatory challenges in their children (Choe et al., 2013; Wolford et al., 2019). Depressed parents also might have impaired parental mentalizing, which lays the foundation for attachment security (Schultheis et al., 2019). Third, ER is influenced by particular parenting practices and behaviours linked to emotional socialization, including reactivity and understanding of the child's mental state (Morris et al., 2007). Existing findings (e.g. Feng et al., 2008; Liu et al., 2017; Silk et al., 2006) indicate disrupted emotional socialization mechanisms in families with depressed parents. These studies have revealed atypical affective interactions, with depressed parents being less responsive to their children's emotions, and displaying less positive and more negative affect (Liu et al., 2017). However, these studies have not measured parental mentalizing, which is the focus of this study.

### Parental mentalizing

Parental mentalizing is defined as the ability of parents to recognize their children's mental states and to explain and give meaning to their behaviour in terms of thoughts, desires and expectations (Slade, 2005). Research has demonstrated that parental mentalizing comprises three essential reflective functions: pre‐mentalizing modes, certainty about the child's mental states, and interest and curiosity about the child's mental states (Luyten et al., 2017; Rutherford et al., 2013). Pre‐mentalizing modes represent a rejection of or defence against mentalization (i.e. the incapacity to enter one's child's subjective world), as seen by a tendency to make maladaptive attributions about the child. Certainty about mental states indicates a parent's level of confidence in attributing mental states to their child, as well as their awareness of the complexity and privacy of mental states. Interest and curiosity about the child's mental state demonstrate a parent's desire and active curiosity to understand their child's inner world. With that being said, very high scores on these dimensions might indicate hypermentalizing, reflecting parents' failure to recognize the child's opacity of mental states, or an excessive or intrusive interest in the child's mental states (Luyten et al., 2017). While pre‐mentalizing modes are seen as a transdiagnostic risk factor demonstrated in diverse mental health disorders including depression, certainty and interest and curiosity regarding mental state are considered key protective factors that mitigate the adverse consequences of multiple psychopathological conditions (for review, see: Luyten et al., 2020). Therefore, in this study, pre‐mentalizing modes will be examined as a mediating factor in the link between parental depressive symptoms and the child's ER, while certainty and interest and curiosity about mental state will be examined as moderating factors.

### Parental mentalizing and children emotion regulation

Parental mentalizing seems to be a key factor in fostering the ability of ER in young children (see for review: Camoirano, 2017). For example, a recent study revealed that toddlers of mothers with better mentalizing abilities handle distress better by seeking comfort, while toddlers of mothers with lower ability tend to become aggressive (Borelli et al., 2021). Accordingly, maternal mentalizing significantly influences children's ER skills, highlighting its positive impact. Furthermore, it was found that better parental mentalizing predicted better ER skills in their children, via higher parental competence (Gordo et al., 2020) and attachment style (Nijssens et al., 2020). One explanation for these consistent findings is that parents with high mentalizing capacities are better able to assist their children in learning how to make sense of their own mental states, thereby developing self‐mentalizing capacities and ultimately leading to better self‐regulation (Álvarez et al., 2022; Camoirano, 2017; Fonagy & Target, 1997). These findings support our assumptions that mentalizing may mitigate the effects of parental depressive symptoms on subsequent child ER.

### Parental mentalizing and parental depression

Importantly, mentalizing is not merely a trait‐like capacity of an individual; rather, it is dynamic and influenced by the interpersonal context, as well as by the levels of stress and arousal, with very high or very low arousal being linked with a decreased ability to mentalize (Luyten et al., 2020). That is, individuals who experience depressive symptoms might report lower parental mentalizing due to various reasons (Georg et al., 2023). First, depression is related to emotion dysregulation, which has been found to predict greater levels of pre‐mentalizing, that is a non‐mentalizing mode (see for review: Schultheis et al., 2019). Second, depressive symptoms relate to lower self‐esteem and may therefore harm the parent's confidence in their parenting abilities (Dix & Meunier, 2009). A lack of confidence can lead to self‐doubt and hesitation in responding to the child, making it difficult to engage in reflective and attuned parenting practices (Gordo et al., 2020). Moreover, depression is characterized by negative thought patterns, self‐criticism and feelings of worthlessness. These cognitive patterns can impair the parent's judgement and perception of their child's behaviour, making it difficult to understand their child's mental state (Georg et al., 2023).

Although Georg et al. (2023) found that parental mentalizing are not always impaired in depressed parents, with variations depending on the assessment measures used, many studies support the claim that parental depression compromises mentalizing capacities (see for review: Katznelson, 2014). For instance, Ramsauer et al. (2014) concluded that depressed mothers had lower mentalization as they were less likely to reflect on their child's needs and mental states than non‐depressed mothers. Moreover, they showed a lack of understanding and sensitivity towards their child's needs and wishes, and they had difficulties in seeing the child as an independent entity separately from them. Also, Krink et al. (2018) reported that higher levels of depressive symptoms were associated with more pre‐mentalizing modes among mothers. Notably, there was no statistically significant connection between maternal depression and their scores related to interest and curiosity regarding mental states. It is therefore can be inferred that depressive symptoms do not necessarily relate to the degree of mothers' interest and curiosity in understanding their child's inner states, it might be that these specific mentalizing capacities are more stable and trait‐oriented. Instead, depressive symptoms are primarily connected to a reduction in the capacity of mothers to reflect on themselves and others, contributing to a heightened likelihood of distorted perceptions of self and child (Khoshroo & Seyed Mousavi, 2022; Krink et al., 2018).

### The current study

Building upon the literature in this field, it becomes evident that when parents have elevated levels of depressive symptoms, they might demonstrate a lower ability to identify and understand their children's emotions, which subsequently might affect the child's own mentalizing and ER abilities (Georg et al., 2023; Sprecher et al., 2023). These findings led us to examine whether pre‐mentalizing modes will serve as a mediating factor, explaining how parental depression affects the child's ER. Simultaneously, we also tested whether certainty and interest, and curiosity regarding mental states will each play a moderating role, influencing the strength of this relationship. Hence, this study seeks to explore the potential mediating role of parental pre‐mentalizing modes in the connection between parental depressive symptoms and their children's ER skills. Additionally, we aim to investigate the moderating influence of certainty and curiosity in this relationship. Our hypotheses are as follows: (1) Parents with depressive symptoms will lean towards pre‐mentalizing modes, contributing to the child's emotional dysregulation. (2) High parental curiosity and certainty regarding the child's mental state will mitigate the detrimental effects of depressive symptoms on child development.

## METHOD

### Participants

This study was part of a larger study aimed at examining distress symptoms among kindergarten teachers, preschool children and their parents during the COVID‐19 pandemic (see: https://osf.io/qde24/). This paper focusses only on the consequences of parents' characteristics on preschoolers' emotion regulation; therefore, the kindergarten teacher‐related data were not included. The sample size was calculated as follows: First, based on previous studies that examined the Pearson's correlation coefficient (*r* ~ 0.2–0.3) between different variables among parents and the desired sample for measuring these *r* values with a 95% confidence interval, a power analysis was performed at *α* = .05; Power = 0.80. According to this calculation, the desired sample size is about *N* = 193 for *r* = 0.2 and *N* = 84 for *r* = 0.3 (Bujang & Baharum, 2016). The inclusion criteria was being a parent of a child in pre‐school age. Sample in this study consisted of 732 parents (91% females; Mean age = 36.0, SD = 5.7), of children aged 3–7 years (51% females; Mean age = 4.9, SD = 0.9). The majority of the parents (93%) were in a relationship/married, 4% were divorced or separated, 2% were single, and 1% were widows. Parents had average of 2.9 children (SD = 1.4), and 15.5 (SD = 2.4) years of education (which reflects that most of them had B.A.). Our sample demonstrated a representative distribution across the diverse segments of the Israeli population, comprising 87% Jewish parents, 12% Arabic and 1% from other demographic segments. The participants exhibited diverse levels of religiosity, including secular (40%), traditional (24%), religious (32%), ultra‐Orthodox (2%) or other (2%).

### Procedure

This cross‐sectional study was conducted amidst the COVID‐19 pandemic in Israel, a period characterized by heightened levels of depression symptoms among parents (Feinberg et al., 2022). The research encompassed a substantial and diverse sample, comprising parents of preschool‐age children from various sectors and segments of the Israeli population. The research received ethical approval from two committees: the Ethics Committee of the Office of the Chief Scientist at the Israeli Ministry of Education (file number 12077) and the Ethics Committee of the Faculty of Education at the University of Haifa (file number 520/21). Data collection occurred between April and July 2022, spanning from the end of the fifth wave to the peak of the sixth wave of the COVID‐19 pandemic in Israel. During this period, there was a significant increase in the number of people infected in Israel, following the spread of a new variant (Israel Ministry of Health, 2022). The survey was conducted in both Hebrew and Arabic, aligning with the participants' native languages. The study aimed to gather comprehensive data by employing a cluster probability sampling method based on geographical regions established by the Ministry of Education. However, due to a low response rate using the probability sampling method, convenience sampling techniques were also utilized. This involved reaching out to potential participants through social networks, particularly Facebook and WhatsApp. Data collection was conducted anonymously through Qualtrics. Parents who completed the survey received a POP IT toy for their child.

### Measures

*Parental current depressive symptoms*: Depression was assessed using the Patient Health Questionnaire (PHQ‐9; Kroenke et al., 2001), which is a 9‐item self‐report brief diagnostic measure for depression. Importantly, due to ethical considerations, the item concerning suicidal tendencies was omitted from the questionnaire in this study; therefore, the questionnaire included only eight items. Participants were asked about the frequency of their experience with various depressive symptoms over the past 2 weeks (such as ‘Little interest or pleasure in doing things’) on a 4‐point Likert scale, ranging from 0 (not at all) to 3 (nearly every day). The total score, ranging from 0 to 18, reflects the severity of depressive symptoms, with higher scores indicating more pronounced symptoms. Cronbach's alpha coefficient was good (*α* = .89) in this sample.

*Parental mentalizing*: Parental mentalizing was evaluated using the Parental Reflective Functioning Questionnaire (PRFQ; Luyten et al., 2017), an 18‐item self‐report survey scored on a 7‐point Likert scale. The PRFQ consists of three subscales: The first subscale examines certainty about mental states and captures whether parents are excessively certain or uncertain about their child's mental states (e.g.―‘I always know what my child wants’). The second subscale measures interest and curiosity in mental states, reflecting parents' curiosity about their child's emotional states (e.g. ‘I am often interested in understanding how my child feels’). These dimensions can emerge in a maladaptive fashion as hyper‐mentalizing (parents who are overly certain or curious about their child's mental state) or hypo‐mentalizing (parents who lack confidence or interest in their child's mental state). The scoring for predicting high or low scores on the Certainty in Mental States and Interest and Curiosity subscales of the PRFQ has no cut‐offs. This suggests that moderate levels of both subscales may be more optimal, while either low or very high levels may be more dysfunctional. The third one assesses pre‐mentalizing modes, indicating a rejection or defence against mentalizing (e.g.―‘My child sometimes gets sick to keep me from doing what I want to do’). In this study, the Cronbach alphas for pre‐mentalizing modes, certainty about mental states, and interest and curiosity in mental states were acceptable (*α* = .74, *α* = .70, *α* = .82), respectively.

*Children's emotion regulation*: Children's emotion regulation ability was measured with the Emotion Regulation Checklist (ERC; Shields & Cicchetti, 1997) completed by parents. The checklist comprises 24 items that assess the child's regulation abilities (e.g.—‘My child can say when he/she is feeling sad, angry, or mad, fearful or afraid’). Parents rate each item on a 4‐point scale, ranging from 0 (almost never) to 3 (almost always). A total score of emotion regulation, ranging from 0 to 72, was calculated by reversing negatively weighted items and summing the scores on each item, with higher scores indicating more adaptive emotion regulation (Ramsden & Hubbard, 2002). In this study, the Cronbach's alpha coefficient was good (*α* = 0.84).

*COVID‐19‐related stress*: Measured by the COVID‐19 Concerns Questionnaire (Khouri et al., 2022), where parents rated their worry on a Likert scale of 1–5 (1—‘not at all’, 5—‘extremely’). The questionnaire covered health concerns for the participant and their relatives (3 items), economic/employment concerns (3 items), mental and interpersonal well‐being concerns (4 items) and the pandemic's impact on children (3 items). The total score was the average of all 13 items, with an excellent internal reliability of *α* = .90.

### Data analysis

All the analyses were conducted using the SPSS.27 software (Armonk, NY: IBM Corp). First, the means, standard deviations and bivariate correlations among the study variables were investigated. Next, to estimate the indirect effect of parental current depression on child emotion regulation via parental pre‐mentalizing modes as the mediator, we employed the PROCESS mediation macro in SPSS (Hayes, 2017; Model 4). This method directly measures the size of the mediating effects using 5000 bootstrapped samples and allows to perform regression analysis without needing the data to follow a normal distribution. When the confidence intervals of the indirect effect of a mediator do not include 0, it is considered statistically significant. In addition, parental certainty as well as interest and curiosity about their child's mental state were examined as moderators of the link between current depressive symptoms and the child's emotion regulation skills using multivariate linear regression. In the first step, COVID‐19‐related stress was entered as a covariate. Parental depressive symptoms, parental interest and curiosity as well as parents' certainty of the child's mental state were entered in Step 2. Finally, the interactions between parental depressive symptoms and parental mentalizing were entered in the third step to explore the moderating effects. To account for the potential contribution of stress attributed specifically to the COVID‐19 on the outcomes, COVID‐19‐related stress was included as a single covariate in all analyses.

## RESULTS

### Descriptive statistics

Table 1 displays the means and bivariate Pearson's correlation coefficients. There was a positive correlation between current parental depressive symptoms and pre‐mentalizing modes (*r* = 0.31, *p* < .001), as well as a negative correlation between parental depressive symptoms and the child's emotion regulation skills (*r* = −0.23, *p* < .001). Pre‐mentalizing modes exhibited a negative association with the other two reflective functions, namely certainty about mental states (*r* = −0.08, *p* < .05) and interest and curiosity about mental states (*r* = −0.09, *p* < .05), as well as with the child's emotion regulation skills (*r* = −0.47, *p* < .001). Additionally, certainty about mental states and interest and curiosity about mental states were positively correlated with each other (*r* = 0.25, *p* < .001) and with the child's emotion regulation abilities (*r* = 0.25, *p* < .001; *r* = 0.11, *p* < .01, respectively).

**TABLE 1:** Descriptive statistics.

	Mean (SD)	1	2	3	4
1. Parental depressive symptoms	5.4 (5.3)				
2. Pre‐mentalizing modes	1.9 (0.9)	0.31***			
3. Certainty about mental states	4.5 (1.1)	−0.06	−0.08*		
4. Interest and curiosity	5.6 (0.9)	0.04	−0.09*	0.25***	
5. Child ER	54.8 (8.5)	−0.23***	−0.47***	0.25***	0.11**

Abbreviation: SD, standard deviation.

* *p* < .05.

** *p* < .01.

*** *p* < .001.

### Mediation analyses

**FIGURE 1:**
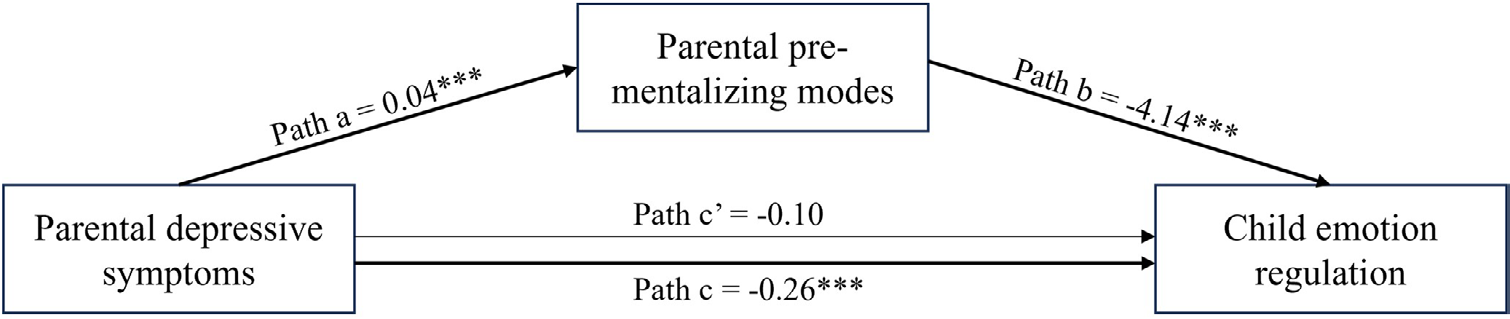
The mediating role of pre‐mentalizing modes between parental depressive symptoms and child ER, accounting for COVID‐19‐related stress. All coefficients are unstandardized. ***p < .001; bold lines represent significant paths.

Model 4 in the SPSS PROCESS macro was used to test the mediation effect of parental pre‐mentalizing modes on the link between parental current depressive symptoms and child ER skills, with COVID‐19‐related stress as the covariate (Figure 1). Results are presented in Table 2. The model explained 23.5% of the variance in child ER (*F* (3,728) = 74.33, *p* < .001). Supporting our hypothesis, the study found a direct effect of parental depression on the child's ER skills, so children of parents who reported higher depressive symptoms had lower ER skills, as indicated by parents' reports (total effect; *B* = −0.25, *t* = −3.94, *p* < .001). When the parental pre‐mentalizing mode was included in the analysis as a mediator, this coefficient was no longer statistically significant (direct effect; *B* = −0.10, *t* = −1.70, *p* = .09). The direct link between parental current depressive symptoms and parental pre‐mentalizing modes was positive and significant (*B* = 0.04, *t* = 5.63, *p* < .001), suggesting that when parental depressive symptoms are high, so is the tendency of parents to exhibit pre‐mentalizing modes. Next, parental pre‐mentalizing modes exhibited a direct link to the child's ER skills (*B* = −4.14, *t* = −12.50, *p* < .001). Finally, the indirect link between parental depressive symptoms and the child's ER via pre‐mentalizing modes was significant (*B* = −0.15, *p* < .001). COVID‐19‐related stress was not a significant predictor of the child's ER (*B* = −0.59, *t* = −1.78, *p* = .08).

**TABLE 2:** Total, direct and indirect effects of the mediation model.

	*b*	SE	95% CI	
			Boot LLCI	Boot ULCI
Depression → PM (path a)	0.04***	0.01	0.02	0.05
PM → Child ER (path b)	−4.14***	0.33	−4.79	−3.50
Total effect (path c)	−0.26***	0.07	−0.39	−0.13
Direct effect (Path c′)	−0.10	0.06	−0.22	0.02
Indirect effect				
Depression → PM → Child ER	−0.15***	0.04	−0.23	−0.09

Abbreviations: ER, emotion regulation; PM, pre‐mentalizing modes.

*** *p* < .001.

### Moderation analyses

To test for a potential moderating effect of parental mentalizing on the link between current parental depressive symptoms and a child's emotion regulation skills, we conducted a multivariate regression analysis, with the child's ER skills serving as the dependent variable. The results of these analyses are presented in Table 3.

**TABLE 3:** Moderation analyses.

	*R* ^2^	*F*	*B*	SE	*β*	*t*
*Model 1*	0.5	38.7***				
COVID‐19‐related stress			1.96	0.32	−0.22	−6.22***
*Model 2*	0.13	27.28***				
COVID‐19‐related stress			−1.39	0.35	−0.16	−4.00***
Depression			−0.23	0.06	−0.15	−3.66***
Parental CM			1.62	0.27	0.22	6.06***
Parental IC			0.66	0.33	0.07	2.04*
*Model 3*	0.14	19.71***				
COVID‐19‐related stress			−1.43	0.35	−0.16	−4.14***
Depression			−1.23	0.38	−0.76	−3.22***
Parental CM			1.08	0.38	0.15	2.85**
Parental IC			0.22	0.45	0.02	0.48
Depression × CM			0.10	0.05	0.30	2.00*
Depression × IC			0.09	0.06	0.34	1.49

Abbreviations: CM, certainty about mental states; IC, interest and curiosity.

* *p* < .05.

** *p* < .01.

*** *p* < .001.

First, COVID‐19‐related stress seems to predict worse ER skills among the child (*b* = −1.96, *t* = −6.22, *p* < .001). Second, the results showed that parental depressive symptoms predict lower ER skills in the child (*b* = −0.23, *t* = −3.66, *p* < .001). Additionally, parental certainty about mental states and parental interest and curiosity predict higher ER skills in the child (*b* = 1.62, *t* = 6.06, *p* < .001; *b* = .66, *t* = 2.04, *p* < .05, respectively).

**FIGURE 2:**
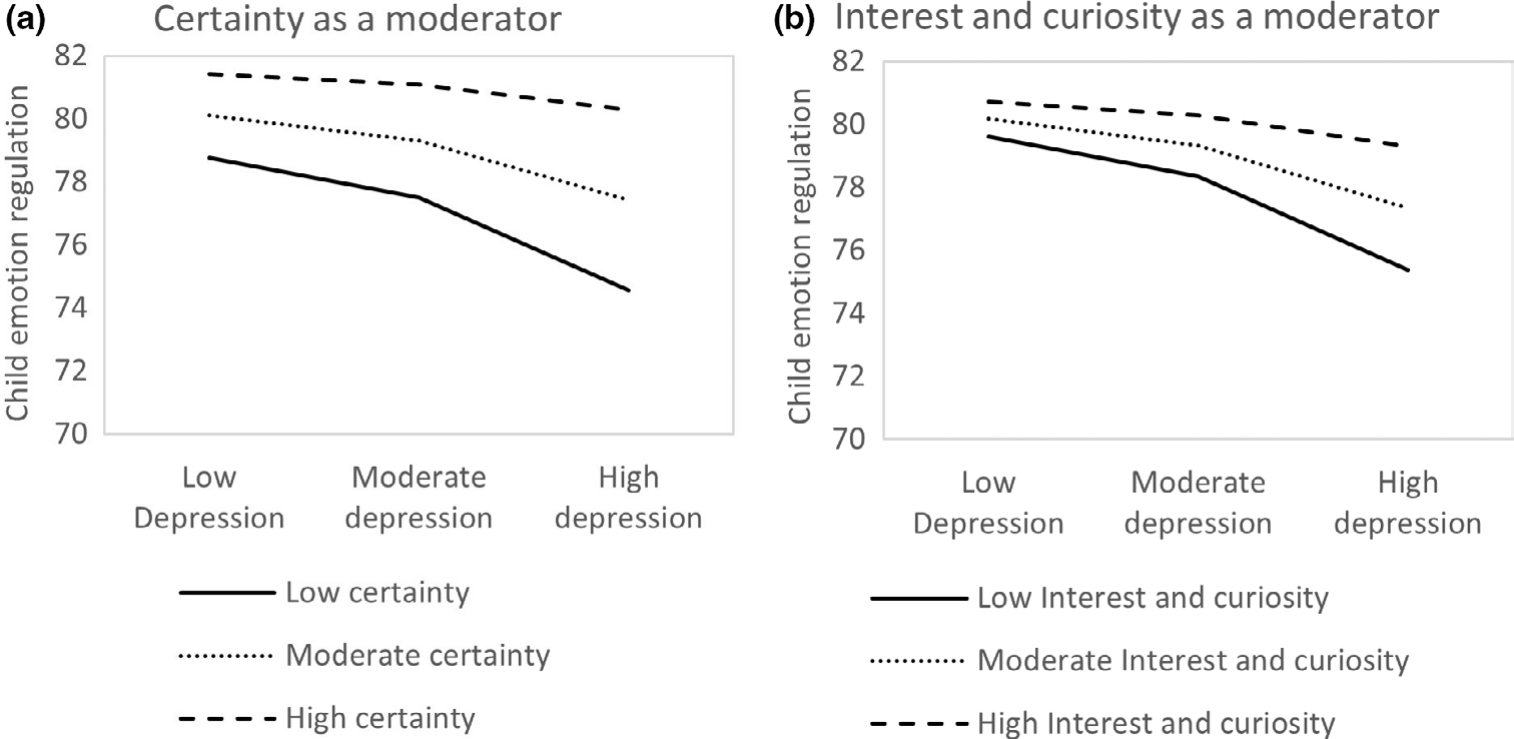
Interaction between paternal depression and parental reflective functions as predictors of the child's emotion regulation. (a) Certainty about mental states as a moderator. (b) Interest and curiosity as a moderator.

For the moderation analysis, the interaction between parental depressive symptoms and parental certainty about mental states significantly predicts the child's ER skills (*b* = .10, *t* = 2.00, *p* < .05). This indicates that higher parental certainty weakens the negative impact of parental depressive symptoms on child ER skills (Figure 2a). However, the interaction between parental depressive symptoms and parental interest and curiosity was not significant (*b* = .09, *t* = 1.49, *p* = .14), indicating that these factors do not moderate the relationship (Figure 2b). This model accounted for 14% of the variance, *F* (6,725) = 19.71, *p* < .001. This finding indicates that parental certainty weakens the link between parental depressive symptoms and child ER skills (see Figure 2a), while the interest and curiosity factor does not (Figure 2b).

## DISCUSSION

The current study's purpose was to explore whether parental pre‐mentalizing modes mediate the link between current parental depressive symptoms and child ER and whether parental interest and curiosity, as well as certainty about the child's mental state, moderate this link. The theoretical framework drawn from Morris et al. (2007) model of emotion socialization guided our investigation into how parental factors, particularly depressive symptoms, contribute to the development of children's ER abilities. As expected, we have found a negative link between parental depressive symptoms and children's ER skills. This link was mediated via a higher tendency of parental pre‐mentalizing modes. Next, consistent with our hypothesis, parental certainty about mental state was found to be a protective factor against the adverse effects of parental depression on child development, as it was associated with a weaker link between parental depressive symptoms and child ER skills. However, in contrast to our expectations, parental interest and curiosity about mental states did not moderate this link.

Consistent with existing literature (Camoirano, 2017; Ghanbari et al., 2023; Sprecher et al., 2023), our study revealed that high levels of both parental certainty and interest and curiosity about mental states demonstrated a significant positive correlation with ER skills among preschoolers. This implies that when parents exhibit greater certainty and curiosity regarding their child's mental state, the likelihood of the child displaying proficient ER skills increases. We also found a significant negative association between parental depressive symptoms and children's ER skills. This finding aligns with previous research demonstrating the impact of parental mental health on children's emotional development (Álvarez et al., 2022; Ghanbari et al., 2023; Gordo et al., 2020). According to the model of emotional socialization (Morris et al., 2007), this link can be explained indirectly by more negative atmosphere within the family, maladaptive parental modelling of ER and reduced tendency to use parenting practices (influenced by mentalizing) that foster ER.

The findings of the present study are in line with this model, as parental pre‐mentalizing modes, which is an important parenting emotion‐related ability, or lack thereof, were found to mediate the link between parental depressive symptoms and the child's ER skills. Accordingly, parents with higher levels of depressive symptoms were more likely to exhibit pre‐mentalizing modes, suggesting a limited ability for mentalizing of the child's mind. Importantly, these pre‐mentalizing modes were found to be directly correlated with children's ER, indicating that parental mentalizing capacities play a crucial mediating role in the transmission of ER skills to children. That is, depression can hinder parents' ability to reflect on their child's mental states, presumably due to cognitive distortions associated with the condition (Georg et al., 2023). Thus, in their interactions with their children, they are consistently more inclined to resort to pre‐mentalizing modes, that are strongly associated with elevated psychological distress. This tendency towards pre‐mentalizing modes may potentially hinder the child's ER abilities, as parents who struggle with adopting a ‘meta‐position’ towards the other's experience exhibit deficiency in using mental states as a reliable source of information, thus experiencing more confusion and difficulty in identifying their child's mental state, and might also exhibit a tendency to experience confusion between self and other (Luyten et al., 2020). In turn, they may find it challenging to assist the child in understanding their own emotional states. These parents may also model less effective ER strategies to their children (Shai et al., 2023), ultimately resulting in the development of less adaptive ER tendencies (Camoirano, 2017; Feng et al., 2008). Noteworthy, however, that this study is cross‐sectional, therefore the current findings cannot establish causality or determine the direction of these relationships.

Previous study revealed that interest and curiosity in mental states, as well as certainty about mental states, were not significantly associated with parental depression (Krink et al., 2018). This suggests that parents' engagement with their child's mental states and their awareness of mental opacity potentially remain unaffected by the severity of their depressive symptoms. On the other hand, the limited ability to mentalize, as indicated by the pre‐mentalizing modes subscale, is linked to depression. Consequently, interest and curiosity in mental states and certainty about mental states were explored as moderating variables presumably capable of mitigating the impact of depression on a child's ER, rather than serving as mediating variables. That is, we explored interest and curiosity in mental states and certainty about mental states as conditions that modify the primary relationship between parental depressive symptoms and the child's ER (moderation) instead as the underlying mechanism driving this relationship (mediation).

Consistent with previous findings (e.g. Ghanbari et al., 2023), our results revealed that parental certainty about mental states weakened the link between parental depressive symptoms and children's ER skills. This suggests that a parent's certainty about their child's mental states may act as a protective factor, mitigating the negative impact of depressive symptoms on children's ER. It is possible that this effect may be at least partially associated with their ability to reflect on both their own mental states and their child's both separately and interdependently. Such reflection could be associated with the parents' ability to take the perspective of their child and support them in adaptively regulating their emotions in a manner that assists their child in learning how to manage their own emotional states (Fonagy & Target, 1997).

In contrast, the interaction between depression and parental interest and curiosity did not reach statistical significance. Accordingly, while it is possible that certainty regarding mental states may provide parents with a sense of competence and security, diminishing the correlation between depression and the child's regulatory abilities, parental interest and curiosity may not necessarily bring a sense of competence to a parent, particularly for those dealing with depression that reduces self‐esteem. Thus, while our results are consistent with previous studies depicting the positive link between parental interest and curiosity and the child ER skills (Álvarez et al., 2022; Ghanbari et al., 2023; Gordo et al., 2020), our hypothesis regarding the moderating effect of parental interest and curiosity on the link between parental depression and child's ER was not confirmed. Further examination of our data showed that the current sample was relatively high in this subscale (reporting a high mean score), with participants reporting levels one standard deviation above the sample average actually reporting nearly maximal levels of this capacity, namely, exhibiting hypermentalizing (Luyten et al., 2017). This might reflect the parent's anxiousness about the child's mind: the parent becomes less available and attentive to the child's actual experience in a way that may be associated with feelings of overwhelm and even intrusiveness. Another possible explanation is that a child in distress or emotionally overwhelmed due to low ER capacities leaves the parent in a state of constant misunderstanding and wondering about the child's mental state. In this situation, the opposite relationship exists, that is, the child's difficulties contribute to the parent's difficulty, which manifests itself in the parents' hypermentalizing (Luyten et al., 2017). Future research could delve deeper into understanding the nuanced interplay between parental mentalizing dimensions and depressive symptoms in shaping children's emotional development.

### Limitations

While our study provides valuable insights, several limitations should be acknowledged. The cross‐sectional design limits our ability to establish causal relationships and longitudinal research is needed to explore the temporal dynamics of these associations and assess the interplay between the study variables over time. For example, as mentalizing is dynamic and influenced by the interpersonal context, it is possible that child ER capacities may impact parents' capacity to mentalize, which in turn might impact parents' depressive symptoms. Utilizing longitudinal, cross‐lagged designs would enable to assess reciprocal effects. However, it is important to highlight that the study employed a sizable and representative sample, encompassing diverse sectors and various socioeconomic strata within the population. A second limitation is related to the reliance on parental self‐report measures, with no child or preschool teachers independent report measures used, which may introduce bias particularly in the context of mentalizing. If parents possess lower mentalizing abilities, their reports on their child's mental state or behaviour (ER tendencies in this case) may be subject to increased bias. Therefore, future studies could benefit from incorporating multi‐method assessments such as interviews and observational measures, such as the Parent Development Interview (PDI; Slade, 2005) to measure parental reflective functions, a preschool teacher report, or a frustration task to measure the child's ER skills (Calkins et al., 1999). Third, we used the PHQ to measure depression, although this scale has good diagnostic properties, it assesses a current depressive episode (Gilbody et al., 2007). Future studies will benefit from using clinically diagnosed depressed parents. Another limitation regarding this scale is that due to ethical considerations the item concerning suicidal tendencies was removed, which might affect the results. Lastly, this study was conducted during the COVID‐19 pandemic, a factor that made it particularly valuable, since parents during this period showed high depression symptoms. Yet, it is important to acknowledge the potential impact of pandemic‐related stress on the study results, potentially leading to lower mentalizing capacities in parents beyond the symptoms of depression. While the researchers introduced COVID‐19‐related stress as a control variable, the broader context of the pandemic could still have influenced the outcomes.

### Conclusions and clinical implications

Our study aimed to investigate the mediating role of parental pre‐mentalizing modes in the link between current parental depressive symptoms and child ER, as well as the moderating role of parental certainty and parental interest and curiosity, on this link. The current study focussed on preschool children, recognizing this stage as pivotal for the development of ER skills (Feng et al., 2008). The research sample, comprehensive and representative, comprised parents from different strata and sectors within the Israeli population. The results of this study contribute to the growing body of literature on preschool children emotional development in the context of parental psychopathology. Understanding the mediating and moderating factors involved in this complex process is essential for informing targeted interventions aimed at promoting children's emotional well‐being, particularly in the presence of parental depressive symptoms. More specifically, the current study highlights the importance of promoting parental mentalizing while working with families, and particularly with parents who are experiencing depressive symptoms that may jeopardize mentalizing capacities. Indeed, a growing body of evidence has demonstrated that targeting parental mentalizing in clinical trials enhanced family functioning and improved parent–child interactions (Byrne et al., 2020; Lo & Wong, 2020; Slade et al., 2020), and that parental mentalizing is linked with more adaptive ER skills and psychological functioning among parents and their children (Camoirano, 2017; Shai et al., 2023). Our findings suggest that mentalizing might be particularly relevant in the context of parental depression, as it is not only underlying the link between parental depression and child's ER, but it may also buffer the negative impact of parental depression on child's ER. Thus, we suggest that professionals working therapeutically with families, and particularly with parents who are currently experiencing depressive symptoms may benefit from using therapeutic interventions enhancing parental mentalizing. Specifically, in this context, it is important to encourage parents to adopt a mentalizing stance towards their children, namely, adopt their child's perspective and treat them as a separate psychological agent whose actions are motivated by their own mental states (Hertzmann et al., 2017; Sharp & Fonagy, 2008). This also includes assessing their child behaviour and emotional reactions with curiosity, focusing on perspective‐taking, and reflecting on their own reactions and mental states (Luyten et al., 2017). It would be potentially helpful to assist parents in identifying their tendencies to prementalizing modes (e.g. notice interpretations that might stem from their own depressive mental states and related negativity) and to hypermentalizing (e.g. notice when they are excessively worrying about their child mental state) and recognizing the specific interpersonal context where these tendencies are activated. Thereby, keeping their child in mind and gaining more reflectivity and flexibility in interpreting their child mental states and experience more certainty in their ability to do so. In turn, the capacity to better mentalize their child may assist parents who are dealing with depressive symptoms in being more attuned to their children's needs and regulating their emotions more accurately, thus potentially fostering the child's ER skills (Ghanbari et al., 2023; Hertzmann et al., 2016; Luyten et al., 2020). Moreover, given that parents who are dealing with depressive symptoms might experience difficulties in creating a therapeutic alliance (Kendra et al., 2014), validating parents' perspectives and accepting their reluctances in trusting, while reflecting on their state of mind in a non‐judgemental, mentalizing therapeutic stance, may foster the therapeutic alliance and enhance parental mentalizing. Notably, however, these clinical suggestions should be further examined in future studies, focusing on implementing mentalization‐based interventions among parents who are experiencing depressive symptoms.

## AUTHOR CONTRIBUTIONS

**Mor Keleynikov:** Conceptualization; investigation; writing – original draft; methodology; visualization; writing – review and editing; formal analysis; project administration; resources. **Joy Benatov:** Supervision; conceptualization; investigation; writing – original draft; writing – review and editing. **Dana Lassri:** Supervision; conceptualization; investigation; writing – original draft; writing – review and editing. **Noga Cohen:** Writing – review and editing; conceptualization; investigation; writing – original draft; supervision.

## FUNDING INFORMATION

This project received funding from the Chief Scientist of the Israeli Ministry of Education. The results and conclusions of this study are at the sole responsibility of the researchers. The study design, data collection, data analysis, data interpretation, and report writing were all conducted independently of the funders' influence.

## CONFLICT OF INTEREST STATEMENT

The authors have no conflict of interest to declare.

## ETHICS STATEMENT

This study was approved and reviewed by the Ethics Committee of the Office of the Chief Scientist at the Ministry of Education (file number 12077) and the Ethics Committee of the Faculty of Education at the University of Haifa.

## Data Availability

The data are available upon request from the corresponding author.
